# Supplementary figures and images for: Development of an affordable multiplex quantitative RT-PCR assay for early detection and surveillance of Dengue, Chikungunya, and co-infections from clinical samples in resource-limited settings
Source: PLoS Negl Trop Dis. 2025 Aug 11;19(8):e0013250. doi: 10.1371/journal.pntd.0013250 (PMC12352876; doi:10.1371/journal.pntd.0013250)

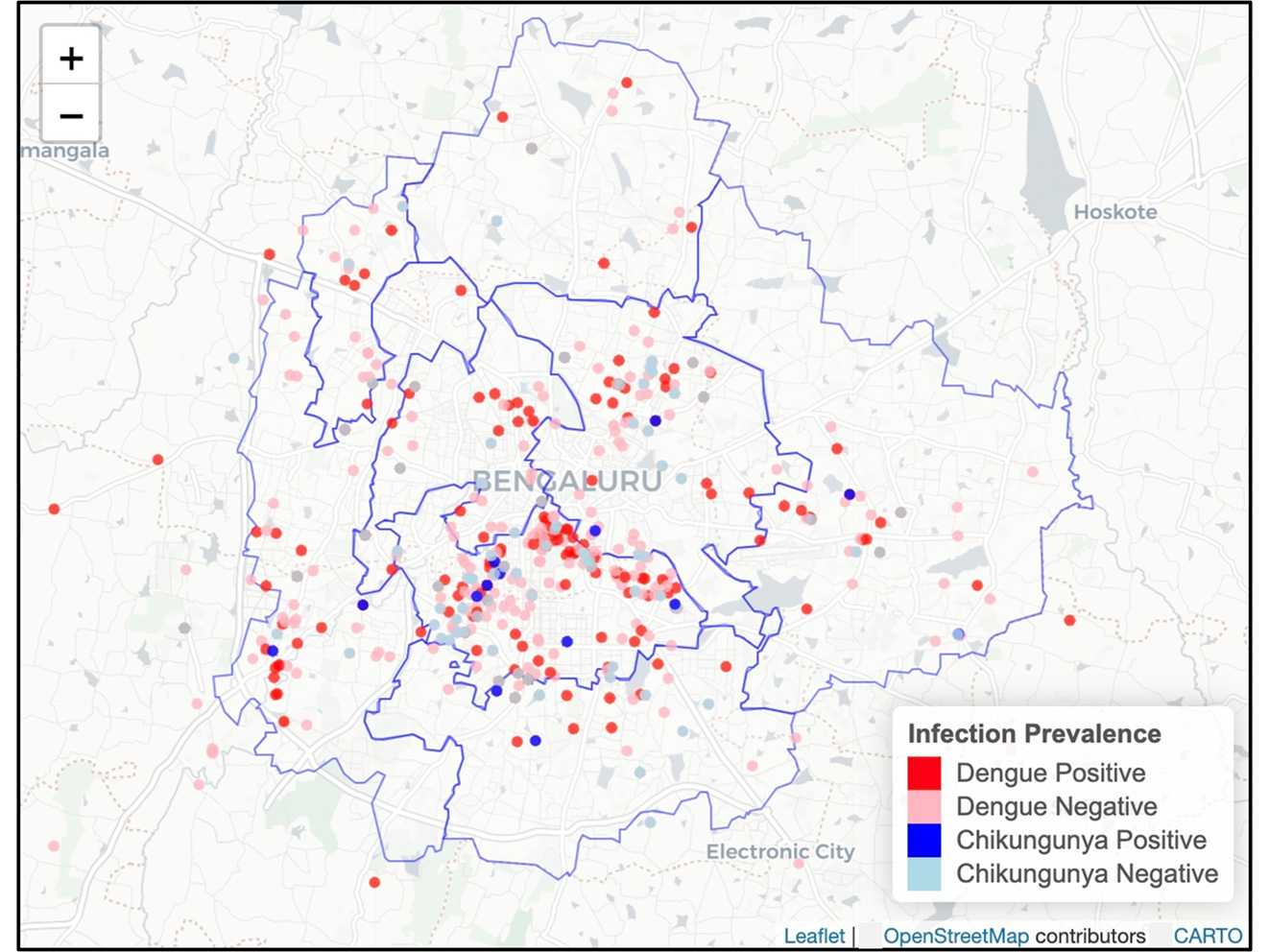

Supplement: S2 Fig — (DOCX) [file pntd.0013250.s006.docx]
