# Supplementary material for: Development of an affordable multiplex quantitative RT-PCR assay for early detection and surveillance of Dengue, Chikungunya, and co-infections from clinical samples in resource-limited settings
Source: PLoS Negl Trop Dis. 2025 Aug 11;19(8):e0013250. doi: 10.1371/journal.pntd.0013250 (PMC12352876; doi:10.1371/journal.pntd.0013250)
Supplement: Attachment — Submitted filename: Reviews on PNTD-D-24-01302.docx [file pntd.0013250.s007.docx]

Review Comments

Title and Abstract:

- Title: The title is clear but could benefit from explicitly mentioning the focus on low-resource settings in terms of both detection and surveillance, not just assay development.
- Abstract (Line 14): Consider briefly mentioning the broader implications of the assay, such as its potential integration into public health programs or its scalability for wider geographic use.

Introduction:

- Line 21-23: It may be helpful to emphasize how misdiagnosis (due to overlapping symptoms) leads to incorrect treatment pathways, especially in low-resource settings where healthcare infrastructure may be limited.
- Line 104-108: The mention of “urbanization” and “human-made habitat” could be expanded. Include more specific examples of how these factors influence Aedes mosquito densities and the spread of arboviruses in different geographical areas.

Methods:

- Line 168-173: Clarify the WHO inclusion criteria used for febrile patients. Including a reference to the guidelines will improve the credibility and reproducibility of the study.
- Line 203-204: Consider providing more details about RNA extraction methods, including quality control procedures, such as RNA integrity testing. This information would enhance confidence in the assay's reliability.
- Line 215: It would be beneficial to explain why the polyprotein genes and E gene were chosen specifically for DENV serotypes and CHIKV, respectively. Explain their diagnostic relevance.

Results:

- Line 320-323: While the DENCHIK assay showed excellent amplification results, provide further context regarding its limit of detection (LoD). How does this LoD compare to that of other similar assays currently in use?
- Line 335-336: The manuscript states that the differences in co-infections were not statistically significant (p = 0.17). Including a discussion of possible reasons for this lack of significance would be useful.
- Line 351-354: Mixed serotype infections are crucial from a clinical management perspective. Expanding this section with literature on how such infections impact treatment or patient outcomes could enrich the discussion.

Discussion:

- Line 495-498: The discussion could delve more into why nucleic acid-based methods (like DENCHIK) are not widely used in resource-poor settings. Is it cost? Infrastructure? A deeper exploration would better justify the importance of a cost-effective solution like DENCHIK.
- Line 503-506: Further elaboration on why current healthcare systems in India are not utilizing such diagnostic tools could create a stronger case for the DENCHIK assay.
- Line 514-518: Mention the limitations of the DENCHIK assay, particularly regarding the 6.65% under-detection of CHIKV infections. What are the potential reasons behind this, and how could it be improved?

Figures and Tables:

- Fig 5 (Line 357-363): The geographical mapping of DENV and CHIKV cases could benefit from further annotations. Adding key demographic or environmental factors to the map could help illustrate hotspots or trends better.
- Table 4 (Line 433): This table discusses sensitivity and specificity, but adding confidence intervals to these values could provide a more precise understanding of the assay's diagnostic accuracy.

Limitations:

- Line 562-563: The limitations section could be expanded to discuss practical challenges in the field, such as sample transport or handling in low-resource settings, which may affect the real-world performance of the DENCHIK assay.

Conclusion:

- Line 552-553: Consider adding a brief recommendation on how the assay could be implemented or scaled for routine use in health facilities, especially for outbreak control and surveillance systems in resource-constrained settings.

Overall:

- Ensure consistent use of abbreviations throughout the document. For instance, both q RT-PCR and qRT-PCR are used, which can cause confusion.
- Ensure all statistical values (p-values, sensitivity, specificity) are accompanied by confidence intervals where relevant, to provide a complete picture of data precision.

These additional suggestions aim to enhance the overall clarity, relevance, and depth of the manuscript. Let me know if you need specific revisions!
