# Supplementary material for: Development of an affordable multiplex quantitative RT-PCR assay for early detection and surveillance of Dengue, Chikungunya, and co-infections from clinical samples in resource-limited settings
Source: PLoS Negl Trop Dis. 2025 Aug 11;19(8):e0013250. doi: 10.1371/journal.pntd.0013250 (PMC12352876; doi:10.1371/journal.pntd.0013250)
Supplement: Attachment — Submitted filename: RESPONSE TO REVIEWERS.docx [file pntd.0013250.s009.docx]

20^th^ February,2024

Mansi Rajendra Malik

Research Scientist

Tata Institute for Genetics and Society

Bengaluru, Karnataka, India

Subject: Response to Reviewers on the manuscript titled, “Development of a cost-effective Multiplex Quantitative RT-PCR assay for early detection and surveillance of Dengue, Chikungunya and Co-infections from clinical samples in Low-Resource settings.

Dear Editors,

**Greetings!**

Thank you for reviewing the manuscript. We have carefully considered all the comments and incorporated them into the revised version. Below, you will find our point-by-point response to each comment.

Additionally, we would like to modify the authorship sequence, as requested by the original first author. Please find attached a screenshot of Dr. Shruthi Uppoor’s email requesting this change.

I hope you will find this manuscript in order and consider it for publication.

Many thanks

Sincerely,

Mansi Rajendra Malik

(on behalf of all co-authors)

**Response to reviewer’s comments**

Title and Abstract:

- Title: The title is clear but could benefit from explicitly mentioning the focus on low-resource settings in terms of both detection and surveillance, not just assay development.
- Response: We have taken note of the comment and changed the title, “Development of an affordable multiplex quantitative RT-PCR assay for early detection and surveillance of Dengue, Chikungunya, and co-infections from clinical samples in resource-limited Settings.”
- Abstract (Line 14): Consider briefly mentioning the broader implications of the assay, such as its potential integration into public health programs or its scalability for wider geographic use.
- Response: We have addressed this point from line number 59-63 in the revised manuscript. The integration and surveillance of molecular assays, such as DENCHIK, will enhance epidemiological monitoring of circulating DENV serotypes, CHIKV, and co-infections. These advancements will provide critical insights for public health authorities, enabling them to prioritize treatment, implement effective control measures, and mitigate the transmission of arboviral infections.
- Introduction:
- Line 21-23: It may be helpful to emphasize how misdiagnosis (due to overlapping symptoms) leads to incorrect treatment pathways, especially in low-resource settings where healthcare infrastructure may be limited.
- Response: This comment has been addressed from line 24-30 in the revised manuscript. The overlapping clinical presentation of both diseases, particularly in early infection, complicates timely and differential diagnosis. In India, diagnosis primarily relies on rapid antigen-based or ELISA-based tests, which are prone to false negatives, leading to underreported disease burden. In resource-limited settings, the absence of confirmatory diagnostics often leads to reliance on clinical symptoms and epidemiological data, increasing the risk of misdiagnosis and undetected co-infections.
- Line 104-108: The mention of “urbanization” and “human-made habitat” could be expanded. Include more specific examples of how these factors influence Aedes mosquito densities and the spread of arboviruses in different geographical areas.
- Response: We have addressed this in lines 103-109 in the revised manuscript.

Increase in Urbanization with built infrastructure (piped water access, water storage) offers a heterogeneous landscape with a gradient of temperature, larval habitats which drives the distribution and abundance of mosquito species. In addition, change in land-use patterns and a diverse gradient of larval habitats such as construction sites, leaking connections among other aquatic habitats created by anthropogenic land use modifications (e.g., bromeliads, Colocasia plants, buckets, plastic containers etc) which are positively associated with the abundance of *Aedes* species

**Methods:**

- Line 168-173: Clarify the WHO inclusion criteria used for febrile patients. Including a reference to the guidelines will improve the credibility and reproducibility of the study.

Response: A total of 903 blood samples were collected from febrile patients meeting WHO criteria, which require a body temperature of ≥38°C and atleast two symptoms such as headache, myalgia, retro-orbital pain, arthralgia, bone pain, or rash.These criteria are particularly relevant for individuals from regions with documented arbovirus circulation, consistent with suspected arboviral infections like dengue and chikungunya.

We have provided the criteria for the inclusion of patients for this study with appropriate references in Line 165-168. Moreover, these are suspected dengue cases which were screened using ELISA as well as molecular assay.

- Line 203-204: Consider providing more details about RNA extraction methods, including quality control procedures, such as RNA integrity testing. This information would enhance confidence in the assay's reliability.

Response: Addressed this query from line 195-201 “Viral RNA was extracted from 140 µL serum samples using the QIAamp Viral RNA Kit (QIAGEN, Hilden, Germany) following the manufacturer’s protocol. The purity of the extracted RNA was assessed spectrophotometrically using the Nanodrop 2000 (ThermoFisher Scientific, USA), with all samples exhibiting an A260/A230 ratio of ~2.0–2.2, indicating minimal reagent contamination. RNA integrity was further evaluated using the TapeStation (Agilent), yielding an RNA Integrity Number (RIN) of ≥7, which is generally considered suitable for q RT-PCR [26]. For q RT-PCR, 25 ng of the extracted viral RNA was used per reaction.”

- Line 215: It would be beneficial to explain why the polyprotein genes and E gene were chosen specifically for DENV serotypes and CHIKV, respectively. Explain their diagnostic relevance.
- Response: Addressed this query from line number 209-215 in the revised manuscript.

Primers covering the Polyprotein genes (poly) were chosen for DENV-1, DENV-2, and DENV-3, as they encode essential information regarding viral population structure, genetic diversity, and pathogenic potential [27–29]. However, the E gene was primarily used for identifying the DENV-2 serotype and Chikungunya virus (CHIKV) due to its role in encoding the envelope protein. This gene exhibits significant antigenic variation among viral strains, making it a crucial marker for serotype differentiation and tracking viral evolution within each virus species [30, 31].

- **Results:**
- Line 320-323: While the DENCHIK assay showed excellent amplification results, provide further context regarding its limit of detection (LoD). How does this LoD compare to that of other similar assays currently in use?
- Response : We have addressed this from lines 344-350 and line 592-598 in the revised manuscript.

The DENCHIK assay showed simultaneous amplification of DENV serotypes and CHIKV AND (LoD) of 10 copies per µL (Fig. 2A, Supplementary Table 3). The in vitro transcribed RNA copy number ranged from 10^1^ to 10^7^ copies per reaction. The coefficient of determination (R^2^) values for DENV-1, DENV-2, DENV-3, DENV-4, and CHIKV were 0.987, 0.987, 0.983, 0.981, and 0.988, respectively, indicating high assay linearity. Additionally, the amplification efficiencies (%Eff) for DENV-1, DENV-2, DENV-3, DENV-4, and CHIKV were 100.3%, 100.2%, 99.8%, 100.5%, and 99.6%, respectively.

- DENCHIK demonstrated a limit of detection (LoD), of approximately ten viral copies per microlitre for both DENV and CHIKV implying that the assay is sensitive for the detection of low viral titers in clinical samples. Notably, LoD of the DENCHIK assay was consistently lower compared to other multiplex q RT-PCR assays, which exhibit detection ranges of 10^1^ to 10^4^ copies per reaction [16, 48]. Furthermore, it outperformed two-tube dengue serotyping assays and standalone Chikungunya assays, which have LoDs ranging from 10^1^ to 10^2^ copies per reaction, underscoring the superior sensitivity of DENCHIK [49, 50].
- Line 335-336: The manuscript states that the differences in co-infections were not statistically significant (p = 0.17). Including a discussion of possible reasons for this lack of significance would be useful.
- Response: Included a discussion pertaining to this query from line 600-606.

Using DENCHIK, we recorded 36% DENV infections, 17% CHIKV infections, and 8% DENV-CHIKV co-infections, which were otherwise underestimated or overestimated when relying solely on ELISA tests. DENCHIK demonstrated significantly higher detection of DENV cases compared to ELISA. However, for CHIKV and DENV-CHIKV co-infections, no significant difference in positivity rates was observed between the two assays. This may be attributed to the persistence of circulating antibodies from past infections, which are detected by ELISA [51, 52].

- Line 351-354: Mixed serotype infections are crucial from a clinical management perspective. Expanding this section with literature on how such infections impact treatment or patient outcomes could enrich the discussion.
- Response: Addressed this comment from line number 649-659 in the revised manuscript. Mixed serotype infections are common in endemic regions, with prevalence ranging from 2.5% to 30% and up to 50% in hyperendemic areas. Evidence suggests that co-infections exacerbate disease severity, with higher incidences of pleural effusion and warning signs, though diarrhea is less frequent in these cases [65]. Secondary dengue infections generally increase disease severity. Mixed serotype infections, particularly those involving DENV-2, are associated with gastrointestinal symptoms and atypical presentations. Secondary immune responses correlate with respiratory symptoms and clinical features of dengue hemorrhagic fever (DHF) and dengue shock syndrome (DSS) [66]. High plasma viral loads in DENV-2 infections further elevate liver enzyme levels and increase DHF/DSS risk in later disease stages [67]. These findings highlight the interplay between serotypes, immune responses, and viral load in shaping clinical outcomes.
- **Discussion:**
- Line 495-498: The discussion could delve more into why nucleic acid-based methods (like DENCHIK) are not widely used in resource-poor settings. Is it cost? Infrastructure? A deeper exploration would better justify the importance of a cost-effective solution like DENCHIK.
- Response: Addresed this query from line number 573-577 in the revised manuscript.Nucleic acid-based assays enable early and accurate detection of DENV and CHIKV; however, the implementation in resource-limited settings is often hindered by high costs, insufficient infrastructure, and a lack of skilled personnel. Additionally, their complex workflow extends turnaround times, while reliance on imported reagents and limited local production further escalates costs and logistical challenges [45].
- Line 503-506: Further elaboration on why current healthcare systems in India are not utilizing such diagnostic tools could create a stronger case for the DENCHIK assay.
- Response: Addressed this comment from line 579-584 in the revised manuscript. India faces similar constraints, with financial limitations, systemic inefficiencies, and a shortage of trained laboratory personnel impeding the routine use of q RT-PCR-based diagnostics for Dengue and Chikungunya. As a result, serology-based methods such as ELISA and rapid antigen tests remain the primary diagnostic tools despite their lower sensitivity. The absence of standardized guidelines and restricted government funding further restrict the large-scale adoption of molecular diagnostics [46, 47].
- Line 514-518: Mention the limitations of the DENCHIK assay, particularly regarding the 6.65% under-detection of CHIKV infections. What are the potential reasons behind this, and how could it be improved?

Response: Addressed this comment from 608-620 in the revised manuscript

DENCHIK identified 7.6% more DENV infections, while IgM ELISA detected 6.65% more CHIKV cases. This variation likely stems from differences in detection methods and the distinct sensitivity and specificity of DENCHIK and ELISA in identifying viral RNA versus antibodies. A study from Manipal, Karnataka [53], reported IgM antibody persistence for up to 10 months post-CHIKV infection, potentially affecting diagnostic accuracy.

q RT-PCR-based assays like DENCHIK exhibit high sensitivity in detecting DENV and CHIKV during the early acute phase when viral RNA is abundant. However, as viremia declines beyond days 8–10, q RT-PCR sensitivity decreases, making ELISA more effective in identifying circulating antibodies indicative of recent or past infections [18, 54, 55]. This diagnostic shift may introduce challenges in differentiating active from resolved infections, underscoring the limitations of IgM ELISA in early CHIKV and DENV-CHIKV coinfection detection, thus emphasizing the need for molecular diagnostics to improve case identification accuracy.

- **Figures and Tables:**
- Fig 5 (Line 357-363): The geographical mapping of DENV and CHIKV cases could benefit from further annotations. Adding key demographic or environmental factors to the map could help illustrate hotspots or trends better.
- Response: The clinical samples analyzed in this study, along with the prevalence data presented in the graph, were derived from densely populated regions of Bengaluru city. However, to achieve a more comprehensive assessment of infection prevalence across diverse demographic groups, a larger and more representative sample set would have been necessary. Due to constraints in sample availability, we were unable to incorporate and analyze a broader dataset.
- Table 4 (Line 433): This table discusses sensitivity and specificity, but adding confidence intervals to these values could provide a more precise understanding of the assay's diagnostic accuracy.
- Response:Added the confidence interval in all diagnostic accuracy tables 3,4,5A and 5B in the revised manuscript.
- **Limitations:**
- Line 562-563: The limitations section could be expanded to discuss practical challenges in the field, such as sample transport or handling low-resource settings, which may affect the real-world performance of the DENCHIK assay.
- Response:Addressed the comment from line number 695-701 in the revised manuscript
- In low-resource settings, DENCHIK’s performance may be affected by challenges such as cold chain limitations, risking RNA degradation; insufficient laboratory infrastructure, increasing contamination risks; and reagent instability due to supply chain disruptions. Contamination control is hindered by limited processing spaces, while cost and sustainability concerns arise from reagent procurement and equipment maintenance. Weak digital infrastructure further delays data reporting and public health responses. Overcoming these barriers is crucial for effective assay deployment.
- **Conclusion:**
- Line 552-553: Consider adding a brief recommendation on how the assay could be implemented or scaled for routine use in health facilities, especially for outbreak control and surveillance systems in resource-constrained settings.
- Response: Addressed the comment from line number 703-710 in the revised manuscript.In conclusion, integrating molecular diagnostics is vital for accurate disease burden estimation in endemic regions. Establishing decentralized q RT-PCR hubs, standardizing protocols, and linking diagnostics with surveillance networks will enhance outbreak detection and response. Capacity building, awareness campaigns, and policy advocacy are essential for scalability. Collaboration with global stakeholders can support funding, technology transfer, and resource mobilization. DENCHIK offers a promising solution for improving arboviral disease diagnosis, surveillance, and clinical management, particularly in resource-limited settings.

- **Overall:**
- Ensure consistent use of abbreviations throughout the document. For instance, both q RT-PCR and qRT-PCR are used, which can cause confusion.
- Response:We have used q RT-PCR consistently across the manuscript.
- Ensure all statistical values (p-values, sensitivity, specificity) are accompanied by confidence intervals where relevant, to provide a complete picture of data precision.
- Response: We have added confidence intervals to all the p values, sensitivity and specificity across the manuscript.

**Reviewer #1: The authors could increase the number of samples to be studied; compare cytokine levels with a control group without any other infection; explain why they did not use the results of molecular tests but rather serological ones; in my opinion, focus on the immune response as treatment management and not diagnosis.**

Response: In urban public health centers and government hospitals, serological assays are the most commonly utilized diagnostic tools. However, the limited availability of technical expertise and infrastructural resources often hinders the implementation of molecular tests. While utilizing immune response-based treatments necessitates additional testing and may not facilitate early clinical management, the rapid and cost-effective diagnosis of the specific disease is crucial for both epidemiological surveillance and effective clinical management of arboviral infections. Regarding the suggestion to increase the sample size and evaluate cytokine levels, we acknowledge this valuable input and will certainly consider it in our future studies.

**Reviewer #2: (No Response)**

Reviewer#3:

1. Line 124. Perhaps “suboptimal” clinical management?

Response: Addressed in line 119-120

Misdiagnosis often leads to false alarms, underestimation of cases, and suboptimal clinical management of disease[1].

.

2. Please add some information in the introduction about chikungunya diagnostic approach and normal time span of viremia.

Response: Addressed in line 112-119

Both DENV and CHIKV incubation period ranges from 2 days to 10 days. Acute infection can

lead to mild undifferentiated acute febrile illnesses, making it clinically indistinguishable from

other viral, bacterial, and parasitic infectious diseases such as influenza, chikungunya,

leptospirosis, filaria, and malaria. [7–9]. Co-circulation of DENV and CHIKV in the same

region and overlapping symptoms and common clinical presentations make the differential and

accurate diagnosis of DENV and CHIKV challenging. For instance, DENV infection can lead

to dengue shock and hemorrhagic fever, while CHIKV infection has been associated with persistent arthralgia for months after the infection has subsided [10]

3. Line 143-144. Why would the use of ELISA underestimate disease burden? It is not clear from the text in the introduction. If previous studies have implicated that, please describe.

Response: please refer line 608-616

DENCHIK identified 7.6% more DENV infections, while IgM ELISA detected 6.65% more

CHIKV cases. This variation likely stems from differences in detection methods and the

distinct sensitivity and specificity of DENCHIK and ELISA in identifying viral RNA versus

antibodies. A study from Manipal, Karnataka [53], reported IgM antibody persistence for up

to 10 months post-CHIKV infection, potentially affecting diagnostic accuracy.

q RT-PCR-based assays like DENCHIK exhibit high sensitivity in detecting DENV and

CHIKV during the early acute phase when viral RNA is abundant. However, as viremia

declines beyond days 8–10, q RT-PCR sensitivity decreases, making ELISA more effective in

identifying circulating antibodies indicative of recent or past infections [18,54,55]

4. Inclusion criteria is referred to reference 18, which I find inappropriate as this is a study that they themselves refer to WHO criteria – please refer to the correct source.

Response: Addressed using multiple references from [17-20] as mentioned in lines 165-168

A total of 903 blood samples were collected from suspected

febrile patients as per WHO inclusion criteria [17-20]) (e.g., high fever (>37.8°C), myalgia, headache, retroorbital pain, arthralgia, and gastrointestinal disorders) (Supplementary file 1)

5. I could not read the supplemental material – but from the discussion I underatnd the authors also had access to specific symptom data – that is not clear from the method section, and not presented anywhere else. Please consider to explain more.

Response:Yes the data was known. Please refer Supplementary file 1.The importance of knowing days post symptom onset and is explained and addressed in lines 495-519.

In comparison to DENCHIK, NS1 ELISA showed a diagnostic sensitivity of 20% and 19%

for detecting DENV within <5 days and >5 days post-symptom presentation. The specificity

of NS1 ELISA in both categories were 93% and 97% respectively (Table 3).

Upon comparing the diagnostic accuracy of IgM ELISA in comparison to DENCHIK, we

observed the detection sensitivity of ELISA in clinical samples presenting symptoms for <5

days and >5 days to be 23% and 30% for DENV, and 24% and 28% sensitive for CHIKV

respectively. However, ELISA was found to be 74% specific for DENV and 77% and 74 %

for CHIKV in both <5 and >5 days categories of symptom presentation (Table 3).

The detection sensitivity of DENCHIK was compared with NS1 antigen ELISA and IgM

ELISA as reference. DENCHIK exhibited sensitivity and specificity of 65% and 63% and

37.96 and 66.45%, respectively. DENCHIK was found to be 26.57% sensitive and 86.07 %

specific for CHIKV detection as compared to IgM ELISA. Similarly, DENCHIK was evaluated

for detection of DENV and CHIKV in the dataset according to the days of onset of symptoms

(Table 4).

DENCHIK, in comparison to NS1 ELISA, exhibited a sensitivity of 31% and 85% for detection

of DENV in the category of <5 days and >5 days post-symptom presentation. However, the

specificity of DENCHIK in both categories was found to be 76% and 57% respectively (Table 4).

Also consider line 717-719

Our findings demonstrate the efficiency

of DENCHIK, an in-house multiplex qRT-PCR assay capable of detecting DENV, CHIKV,

and co-infections from as early as day one of symptom onset.

6. In line 273 the headline is comparison with elisa, but also include validation against commercial tests, please consider to rephrase headline. And add information about the commercial test. Please also specify how and why the 100 samples were selected.

Response:Please refer the headlines of figure 4,5A and 5B.We selected representative 100 clinical samples for validation based on statistical power, feasibility, and regulatory considerations. This sample size provides a reliable estimate of test performance while ensuring meaningful comparisons and minimizing random variability. It balances resource constraints, as clinical sample collection is time-consuming, costly, and subject to ethical limitations. Regulatory bodies like the FDA, WHO, and CLSI often accept 50–100 samples for preliminary assessments, with larger studies required for final approval. Additionally, 100 samples serve as an initial validation step before expanding to large-scale, multi-center trials.

7. Is the number of days since symptom onset known to the researchers? It does not say in the method section, but becomes evident in line 411. Please explain.

Response: Yes the data was known. Please refer Supplementary file 1.The importance of knowing days post symptom onset and is explained and addressed in lines 499-519.

In comparison to DENCHIK, NS1 ELISA showed a diagnostic sensitivity of 20% and 19%

for detecting DENV within <5 days and >5 days post-symptom presentation. The specificity

of NS1 ELISA in both categories were 93% and 97% respectively (Table 3).

Upon comparing the diagnostic accuracy of IgM ELISA in comparison to DENCHIK, we

observed the detection sensitivity of ELISA in clinical samples presenting symptoms for <5

days and >5 days to be 23% and 30% for DENV, and 24% and 28% sensitive for CHIKV

respectively. However, ELISA was found to be 74% specific for DENV and 77% and 74 %

for CHIKV in both <5 and >5 days categories of symptom presentation (Table 3).

The detection sensitivity of DENCHIK was compared with NS1 antigen ELISA and IgM

ELISA as reference. DENCHIK exhibited sensitivity and specificity of 65% and 63% and

37.96 and 66.45%, respectively. DENCHIK was found to be 26.57% sensitive and 86.07 %

specific for CHIKV detection as compared to IgM ELISA. Similarly, DENCHIK was evaluated

for detection of DENV and CHIKV in the dataset according to the days of onset of symptoms

(Table 4).

DENCHIK, in comparison to NS1 ELISA, exhibited a sensitivity of 31% and 85% for detection

of DENV in the category of <5 days and >5 days post-symptom presentation. However, the

specificity of DENCHIK in both categories was found to be 76% and 57% respectively (Table

4).

8. Why is a significant or non-significant difference between DENCHIK and IgM of interest? Too me the interesting question regards true positive or negative test results - sensitivity and specificity. As A PCR would be positive in a different timespan compared to serology, I do not understand these analyses (line 330-336).

Response: Chi-square analysis is a statistical method frequently employed to evaluate the concordance between two diagnostic assays, such as ELISA (Enzyme-Linked Immunosorbent Assay) and PCR (Polymerase Chain Reaction), in the detection of infections or diseases. This analysis determines whether the observed differences in detection rates between these methods are statistically significant or attributable to random variation.

Rationale for Comparing ELISA and PCR is that ELISA detects antibodies or antigens, making it valuable for identifying past or ongoing infections.However, PCR detects the genetic material of a pathogen, offering high sensitivity and specificity in infection diagnosis.

Chi-square analysis facilitates the assessment of detection rate discrepancies between these diagnostic tools, providing insights into their relative performance and potential limitations.

Also refer line 600-620

Using DENCHIK, we recorded 36% DENV infections, 17% CHIKV infections, and 8%

DENV-CHIKV co-infections, which were otherwise underestimated or overestimated when

relying solely on ELISA tests. DENCHIK demonstrated significantly higher detection of DENV cases compared to ELISA. However, for CHIKV and DENV-CHIKV co-infections, no significant difference in positivity rates was observed between the two assays. This may be attributed to the persistence of circulating antibodies from past infections, which are detected by ELISA [51, 52].

DENCHIK identified 7.6% more DENV infections, while IgM ELISA detected 6.65% more

CHIKV cases. This variation likely stems from differences in detection methods and the

distinct sensitivity and specificity of DENCHIK and ELISA in identifying viral RNA versus

antibodies. A study from Manipal, Karnataka [53], reported IgM antibody persistence for up

to 10 months post-CHIKV infection, potentially affecting diagnostic accuracy.

q RT-PCR-based assays like DENCHIK exhibit high sensitivity in detecting DENV and

CHIKV during the early acute phase when viral RNA is abundant. However, as viremia

declines beyond days 8–10, q RT-PCR sensitivity decreases, making ELISA more effective in

identifying circulating antibodies indicative of recent or past infections [18, 54, 55]. This

diagnostic shift may introduce challenges in differentiating active from resolved infections,

underscoring the limitations of IgM ELISA in early CHIKV and DENV-CHIKV coinfection

detection, thus emphasizing the need for molecular diagnostics to improve case identification

accuracy.

9. What do the authors mean with this sentence (line 349-350)?. “There was no significant difference observed in the prevalence of DENV and CHIKV amongst the screened clinical samples”. Is this non statistical difference of importance? What does it tell the reader?

Response: Addressed in lines 602-606

DENCHIK demonstrated significantly higher detection of DENV cases compared to ELISA. However, for CHIKV and DENV-CHIKV co-infections, no significant difference in positivity rates was observed between the two assays. This may be attributed to the persistence of circulating antibodies from past infections, which are detected by ELISA [51, 52].

10. In my opinion the proportion of mixed serotype infection is very high. With an in-house PCR assay this is concerning as it might signify PCR non-specificity. What do the authors think about this problem and was any confirmatory test performed, or sequencing performed? How did the commercial tests performed in the mixed infection group?

Response: Currently, no commercially available assays can simultaneously detect all four serotypes. However, serotype confirmation was achieved using a two-tube assay (Altona Diagnostics). Our analysis demonstrated over 98% concordance in serotype detection when using commercial assays (see Tables 5A and 5B).

11. Please expand the information about mixed infection. I can not fully understand the sentence at line 353: Mixed DENV serotype infections with D2, D3, and D4 serotypes were observed in 112 samples (12.4 %). Did all mixed infections just concern D2, D3 and D4 – and no mixed infection with D1? Were there any mixed infections with 3 serotypes?

Response:There were mixed infections found with different combinations of two ,three and four serotypes involving all the D1,D2,D3 and D4 serotypes.

Please refer lines 397-400

We found DENV1 (D1) serotype as the most prevalent serotype (104/903;11.5%) followed by

DENV2 (D2) (54/903; 5.8%), DENV3 (D3) (32/903;3.5%) and DENV4 (D4) (29/903;3.2%).

Mixed DENV serotype infections with D1, D2, D3, and D4 serotypes were observed in 112 samples (12.4 %).

12. It is likewise surprising that there is a larger difference between the results of the DENCHIK assay compared with NS1 than for IgM as IgM would come later, with less viremia expected.

Response:Addressed in lines 608-629

DENCHIK identified 7.6% more DENV infections, while IgM ELISA detected 6.65% more

CHIKV cases. This variation likely stems from differences in detection methods and the

distinct sensitivity and specificity of DENCHIK and ELISA in identifying viral RNA versus

antibodies. A study from Manipal, Karnataka [53], reported IgM antibody persistence for up

to 10 months post-CHIKV infection, potentially affecting diagnostic accuracy.

q RT-PCR-based assays like DENCHIK exhibit high sensitivity in detecting DENV and

CHIKV during the early acute phase when viral RNA is abundant. However, as viremia

declines beyond days 8–10, q RT-PCR sensitivity decreases, making ELISA more effective in

identifying circulating antibodies indicative of recent or past infections [18, 54, 55]. This

diagnostic shift may introduce challenges in differentiating active from resolved infections,

underscoring the limitations of IgM ELISA in early CHIKV and DENV-CHIKV coinfection

detection, thus emphasizing the need for molecular diagnostics to improve case identification

accuracy.

Higher detection and prevalence of DENV and CHIKV infections by both ELISA and

DENCHIK were observed from June 2022 to September 2022, coinciding with the peak

monsoon season in Bengaluru. This observation aligns with the findings of Dharmamuthuraja

et al. [4] wherein a similar pattern for Aedes mosquito larval habitat was observed coinciding with the peak in Dengue incidence in Bengaluru], correlating increased Aedes mosquito breeding with seasonal dengue incidence. Additionally DENCHIK detected a higher prevalence of DENV infections from October 2022 to December 2022, highlighting its superior sensitivity.

13. Fig 4. Please expand the explanation of the different boxes. At least Box D is not evident.

Response: Addressed in line 364-370

Using the DENCHIK assay, the DENV prevalence of 36.54% was significantly higher

compared to 9% using NS1 ELISA (χ2=20.58, p<0.0001). However, DENCHIK showed no significant difference (23.9%) using IgM (χ2=1.54, p=0.0641). Similarly, DENCHIK assay (17%) showed no significant difference in CHIKV infections with IgM (23%) (χ2=1.125,p=0.28). We found a similar pattern in the detection of co-infections with DENV (14%) and CHIKV (8%) using both NS1 and IgM ELISA respectively, for the detection of Dengue and Chikungunya, and DENCHIK assay (χ2=1.83, p=0.17) (Fig 4).

14. In table 5 a and be, please spell out w.r.t

Response: Addressed and replaced the headings of table 5A and table 5B

15. Please consider to move the section about a comparison with commercial test to the beginning of the result section. Please also add information, in introduction and or discussion about previous validation of the commercial tests – to give the reader an possibility to determine the appropriateness of their use as a gold standard.

Response: Addressed in lines 686-693

Compared to commercially available q RT-PCR and serotyping kits, DENCHIK exhibited

>98% sensitivity and specificity for all four DENV serotypes and CHIKV, establishing it as a

highly reliable molecular diagnostic tool. Additionally, DENCHIK enabled the detection of

DENV-CHIKV co-infections with 96% sensitivity and 98% specificity. Across the four

DENV serotypes, the assay maintained >90% sensitivity and specificity. Furthermore,

DENCHIK demonstrated high diagnostic accuracy in clinical samples collected within and

beyond five days of symptom onset, outperforming NS1 and IgM ELISA tests, which exhibit

phase-dependent diagnostic limitations [69, 70].

16. In all tables there are columns of LR, OR, RR and AR. How should the reader interpret all these numbers? I would prefer to see the actual number of tests instead, and number of mismatches.

Response:LR,OR and AR are diagnostic accuracy parameters ,however sensitivity and specificity between two tests has been addressed in detail in lines 499-519.

Additionally please refer table 4,5A and 5B

In comparison to DENCHIK, NS1 ELISA showed a diagnostic sensitivity of 20% and 19%

for detecting DENV within <5 days and >5 days post-symptom presentation. The specificity

of NS1 ELISA in both categories were 93% and 97% respectively (Table 3).

Upon comparing the diagnostic accuracy of IgM ELISA in comparison to DENCHIK, we

observed the detection sensitivity of ELISA in clinical samples presenting symptoms for <5

days and >5 days to be 23% and 30% for DENV, and 24% and 28% sensitive for CHIKV

respectively. However, ELISA was found to be 74% specific for DENV and 77% and 74 %

for CHIKV in both <5 and >5 days categories of symptom presentation (Table 3).

The detection sensitivity of DENCHIK was compared with NS1 antigen ELISA and IgM

ELISA as reference. DENCHIK exhibited sensitivity and specificity of 65% and 63% and

37.96 and 66.45%, respectively. DENCHIK was found to be 26.57% sensitive and 86.07 %

specific for CHIKV detection as compared to IgM ELISA. Similarly, DENCHIK was evaluated

for detection of DENV and CHIKV in the dataset according to the days of onset of symptoms

(Table 4).

DENCHIK, in comparison to NS1 ELISA, exhibited a sensitivity of 31% and 85% for detection

of DENV in the category of <5 days and >5 days post-symptom presentation. However, the

specificity of DENCHIK in both categories was found to be 76% and 57% respectively (Table

4).

17. The very high prevenlence of DENV and proportion of mixed infection is not discussed. Please consider.

Response: Addressed in lines 646-659

Among mixed serotype infections, DENV-1: DENV-2 was the most prevalent, consistent with

previous major outbreaks in Nepal [63] and Mexico [64].

Mixed serotype infections are common in endemic regions, with prevalence ranging from

2.5% to 30% and up to 50% in hyperendemic areas. Evidence suggests that co-infections

exacerbate disease severity, with higher incidences of pleural effusion and warning signs,

though diarrhoea is less frequent in these cases [65]. Secondary dengue infections generally

increase disease severity. Mixed serotype infections, particularly those involving DENV-2,

are associated with gastrointestinal symptoms and atypical presentations. Secondary immune

responses correlate with respiratory symptoms and clinical features of dengue haemorrhagic

fever (DHF) and dengue shock syndrome (DSS) [66]. High plasma viral loads in DENV-2

infections further elevate liver enzyme levels and increase DHF/DSS risk in later disease

stages [67]. These findings highlight the interplay between serotypes, immune responses, and

viral load in shaping clinical outcomes.

18. There is no discussion about possible limitations to the study methodology, which is commonly included in a discussion section. Please consider to add this and a discussion about generalizability.

Response: Addressed in lines 695-701

In low-resource settings, DENCHIK’s performance may be affected by challenges such as cold chain limitations, risking RNA degradation; insufficient laboratory infrastructure, increasing contamination risks; and reagent instability due to supply chain disruptions. Contamination control may be hindered by limited processing spaces, while cost and sustainability concerns arise from reagent procurement and equipment maintenance. Weak

digital infrastructure could further delay data reporting and public health responses. Overcoming these barriers is crucial for effective assay deployment.
