# Supplementary material for: Development of an affordable multiplex quantitative RT-PCR assay for early detection and surveillance of Dengue, Chikungunya, and co-infections from clinical samples in resource-limited settings
Source: PLoS Negl Trop Dis. 2025 Aug 11;19(8):e0013250. doi: 10.1371/journal.pntd.0013250 (PMC12352876; doi:10.1371/journal.pntd.0013250)
Supplement: Attachment — Submitted filename: RESPONSE TO REVIEWERS-JUNE 2025.docx [file pntd.0013250.s010.docx]

Response to reviewers’ comments

Dear Editors,

Thank you for your positive response on the manuscript. We have revised the manuscript as per reviewers’ suggestions Please find below point-by-point response.

1) *Table 1a and 1b. Consider move to supplement.*

**Response: We have moved Table 1a and 1b to supplementary Table 4A and 4B. .**

2) *There is an unnamed table of test possibilities which I consider unnessesary.*

**Response: We have removed the table and added an appropriate reference. Please refer to lines 293-306 of the modified manuscript.**

3) *Fig2. Consider is all these figures are necessary in the article or if some could be transferred to supplement. Usually only the C option is shown.*

**Response: We have added the assay figure and amplification plots to the supplementary (see Supplementary Fig 1 ).**

4) *Fig 4.. What is in box D is coinfection with dengue and chikungunya, but it is not evident. Please consider to explain better within the figure.*

**Response: We have edited the figure 4 with checkered bar plots and legends that indicate Dengue-Chikungunya co-infection prevalence with respect to both NS1 and IgM ELISA and DENCHIK.**

5) *Fig 5. Please explain more what is and was is not included in each staple. DEV all dengue PCR positive? DENV1 all dengue serogroup 1 positive – or only non-mixed dengue infections? It is not clear what is included.*

**Response:**

**Figure 5 was plotted based solely on the DENCHIK assay, excluding ELISA results. Please refer to the explanation from lines 355-367 in the modified manuscript.**

6) *Fig 6. Is the map needed for the reader? Consider move to supplement. I guess it is living address that is depicted?*

**Response:**

**We have moved the map to Supplementary Fig 2.**

7) *Table 2. What are the percentages? Percentage of what? (and reduce number of deceimals)*

**Response:**

**Table 2 presents the percentage positivity of DENV serotypes, CHIKV, and mixed serotypes by age groups (0–10, 11–20, 21–30, 31–40, 41–50, 51–60, and over 60 years), genders (male and female), and symptoms. All values are rounded to two decimal places.**

8) *Fig 7 and 8. What are the lines? Derived tendencies? (is a tendency-line appropriate?) What are the dots in the upper and lower margins? Are the months correctly placed?* –

**Response: We have updated the figure. Please refer to figures 6 and 7. Each dot represents the mean prevalence with 95% confidence intervals, reflecting different detection methods: NS1 ELISA, IgM ELISA, and qRT-PCR (DENCHIK) for DENV and CHIKV across various months and age groups.**

9) *Fig 9. Selected months – instead of across months. Please consider to have the months in correct order. It says in the text (line 474-475) that detection of Dengue was lowest in September – but with a positive association to NS1. The latter part can been seen in the figure, but not that detection was lowest in septemper. It is stated in the method section that the study was conducted from July to December 2022. Why is June included but not August?*

**Response: We have removed the June data points as the study considered the July 2022 to December period. Please refer to Fig 8 from the modified manuscript for the correct arrangement of all months and see lines 381-416 for the interpretation.**

10) *Table 3 and Table 4. I still miss the actual numbers in these tables – please consider again. Please spell out 95% CI also in the sensitivity and specificity columns. I still question the use of LR, OR, RR, AR as this data is not referred to anywhere in the text, and it is difficult to interpret this data in context.
    Table 5A and 5B. See Table 3-4 about LR, DOR, RR, AR.*

**Response:We have spelled out 95% CI in Tables 2,3,4A and 4B.The significance of LR, OR, RR, and AR has been highlighted from line 293 to 306; 444 TO 459and from line 493 to 506 in the modified manuscript.**

11) *My question about the samples selected for validation against commercial tests was not about why 100 was choses – but how and which samples were selected for the comparison. Random?, consecutive? Or chosen according to result.*

**Response: We randomly selected 100 clinically positive samples validated against the commercial tests. Please refer to lines 475-479.**

Sincerely,

(on-behalf of co-authors)

Mansi Rajendra Malik
